# Dr. Melancthon Storrs

**Published:** 1900-07

**Authors:** 


Dr. Melancthon Storrs, of Hartford, Conn, died at his home in that city, June 9, 1900, aged 76 years. Dr. Storrs was one of the most celebrated surgeons of his time, served with credit in that capacity in the Civil War, worked up to the last moment, and died of sepsis received at an operation. His loss is deeply regretted by a large acquaintance. He was an honorary Fellow of the American Association of Obstetricians and Gynecologists.

